# Atomistic Simulation of the Ion-Assisted Deposition of Silicon Dioxide Thin Films

**DOI:** 10.3390/nano12183242

**Published:** 2022-09-19

**Authors:** F. V. Grigoriev, V. B. Sulimov, A. V. Tikhonravov

**Affiliations:** 1 Research Computing Center, M.V. Lomonosov Moscow State University, Leninskie Gory, 119234 Moscow, Russia; 2 Moscow Center for Fundamental and Applied Mathematics, M.V. Lomonosov Moscow State University, Leninskie Gory, 119234 Moscow, Russia

**Keywords:** molecular dynamics, ion-assisted deposition, thin films, silicon dioxide films

## Abstract

A systematic study of the most significant parameters of the ion-assisted deposited silicon dioxide films is carried out using the classical molecular dynamics method. The energy of the deposited silicon and oxygen atoms corresponds to the thermal evaporation of the target; the energy of the assisting oxygen ions is 100 eV. It is found that an increase in the flow of assisting ions to approximately 10% of the flow of deposited atoms leads to an increase in density and refractive index by 0.5 g/cm^3^ and 0.1, respectively. A further increase in the flux of assisting ions slightly affects the film density and density profile. The concentration of point defects, which affect the optical properties of the films, and stressed structural rings with two or three silicon atoms noticeably decrease with an increase in the flux of assisting ions. The film growth rate somewhat decreases with an increase in the assisting ions flux. The dependence of the surface roughness on the assisting ions flux is investigated. The anisotropy of the deposited films, due to the difference in the directions of motion of the deposited atoms and assisting ions, is estimated using the effective medium approach.

## 1. Introduction

Ion-assisted deposition (IAD) is widely used to improve the quality of films obtained by low-energy deposition processes such as thermal evaporation [1,2,3]. In IAD, the growing film is bombarded by the ions having energy on the order of tens and hundreds of eV. This method ensures the fabrication of dense thin films with low stresses [4], improved laser damage resistance [5], and low absorption in the mid-infrared wavelength range [6].

Structural, mechanical, and optical properties of thin films fabricated using IAD depend on the deposition parameters, such as the energy of the assisting ions, the direction of the ions beam, the intensity of the ions flux, and so on. To investigate these dependencies, various methods of mathematical simulation of the deposition process were used. In [7], the transport equations were applied to study the implantation of N_2_^+^ in metal films. Based on the simulation results, the optimal IAD parameters, including ion current, dose, and deposition rate, were chosen to improve mechanical properties of the growing film [7]. In [8], based on the results of the MD simulation of IAD, a model was developed for the calculation of the surface smoothing and other structural parameters. The formation of the crystallographic texture of polycrystalline films fabricated by the IAD was studied using MD simulation [9]. In [9], the Lennard-Jones pair potential was used for the calculation of the potential energy of atoms in a growing film, while for the interaction of the film atoms with bombarding ions, the electrostatic component was taken into account. It was found in [9] that the sputtering of atoms from the surface and ion-induced damage decrease if the ion beam is oriented along channeling directions of the crystals. In [10], the MD simulation of silicon epitaxy followed by assisting of argon atoms was performed. It was found that the presence of an ion beam leads to a decrease in the temperature of the substrate, at which a high-quality crystal structure is formed [10]. The MD simulation of the IAD of the Cu films was performed in [11]. The energy of the assisting ions was varied from 10 eV to 40 eV. It was found that an increase in the incident angle or an increase in the ionization coefficient leads to a decrease in the surface roughness, and this effect depends on the energy of the assisting ions. The IAD of the amorphous TiO_2_ thin film was studied using the MD method in [12]. The pairwise MA (Matsui, Akaogi) potential [13] was used to calculate the potential energy of interatomic interaction in the film. The energy of the assisting ions was varied from 50 eV to 200 eV. It was found in [12] that the beam of the Ar ions significantly affects the surface roughness and density of the growing film.

As follows from the above review, classical atomistic modeling makes it possible to simulate the IAD of various thin films and calculate the structural parameters of growing films. In our previous works [14,15], an efficient MD-based method for simulating the deposition of dielectric thin films was developed. This method is focused on the use of high-performance computing and makes it possible to simulate the deposition of thin films with technologically significant dimensions. In the present paper, for the first time, the full-atomistic MD is applied for a systematic study of the influence of the flow density of assisting ions on the properties of silicon dioxide films using the clusters with a characteristic size of tens of nanometers. In particular, large cluster sizes make it possible to evaluate the effect of ion assisting on the anisotropy of growing films. The choice of silicon dioxide as a material for research is due to the wide use of this material as a material with a low refractive index in all types of optical coatings.

## 2. Model Description

**Figure 1:**
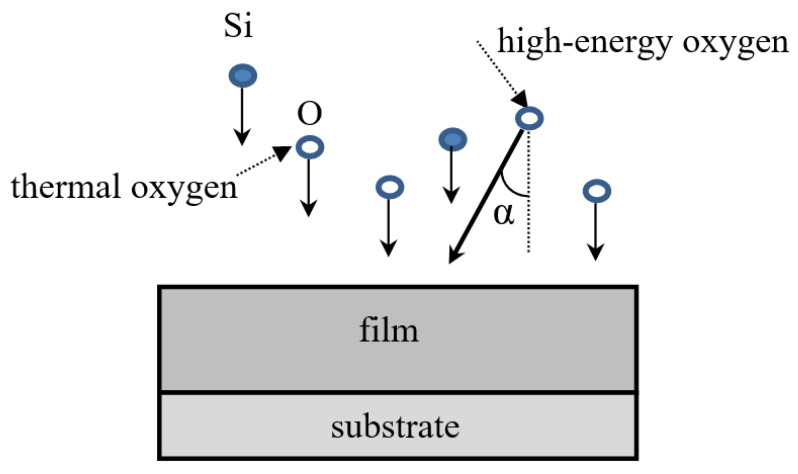
Scheme of the simulation of the ion-assisted deposition.

In the present work, the O_2_^+^ are considered as assisting ions. The MD simulation of IAD of the silicon dioxide films is organized as a sequence of the following steps:

1. Preparing of the fused silica substrate from α-quartz using the melting–quenching procedure [16]. In this work, we use the early prepared substrate [17] with horizontal dimensions of 10 nm × 30 nm and vertical thickness of 6 nm.
2. Insertion of silicon and oxygen atoms with stoichiometric proportion of 1:2 at the top of the simulation box. Each of the oxygen atoms is considered either as thermal or as an assisting ion. Initial coordinates of the atoms are specified randomly. The initial values of kinetic energy are equal to 0.1 eV for Si atoms, 0.1 eV for thermal oxygen atoms, representing the gas in the vacuum chamber, and 100 eV for the assisting ions, having charge +e and mass equivalent to the molar weight of the oxygen molecule, 32 g/mol. The non-electrostatic part of the potential energy of the interaction between assisting ions and other atoms is calculated in the frame of the DESIL force field [14]. The initial velocity of Si atoms and thermal oxygen atoms is directed normally to the substrate. The initial velocity of assisting ions is directed under α = 0 or α = 60° (Figure 1).
3. Start of the MD simulation using the initial conditions described in step 2. Due to the high energy of assisting ions, the time step of MD simulation is equal to 0.05 fs. This step is ten times smaller than that used earlier in the simulation of deposition with an energy of silicon ions of 10 eV. Since velocity increases as the square root of kinetic energy, a tenfold decrease in time step for a tenfold increase in energy seems more than enough. The length of the simulation trajectory is chosen equal to 2 ps. This duration is enough for the assisting atoms to lose their kinetic energy due to collisions with the atoms of the substrate and the previously deposited film layers [14].
4. Continuation of MD simulation with the usual time step of 1 fs. At this stage, the charge of the assisting ions becomes equal to −0.65e, which corresponds to the charge of the oxygen atoms in the frame of the DESIL force field (see below for details), and the mass is equivalent to a molar weight of 16 g/mol. This change in the parameters takes into account that the assisting oxygen ions can be involved in the formation of film layers. The initial state for the simulation in step 4 is the final state of the simulation in step 3. The length of the simulation trajectory is chosen equal to 6 ps, as in our previous simulations [14,15,16,17].
5. The vertical dimension of the simulation box is increased by d = 0.0085 ÷ 0.010 nm to compensate for the growth of film thickness.
6. The injection steps 3–5 are repeated until the film thickness reaches the specified value.

The periodic boundary conditions with NVT (constant number of particles, volume, and temperature) ensemble are used in steps 3 and 4. The Berendsen thermostat [18] is applied to keep the simulation box temperature, T = 300 K, constant. The potential energy of interatomic interaction is calculated using the DESIL force field [14]:

*U* = *q_i_q_j_*/*r_ij_* + *A_ij_*/*r_ij_*^12^ − *B_ij_*/*r_ij_*^6^ (1)

where *q_i(j)_* is the charge of the *i*(*j*)-th atom, *q*_O_ = −0.5*q*_Si_ = −0.65e, *r_ij_* is the interatomic distance, *A*_SiO_ = 4.6·10^−8^ kJ·(nm)^12^/mol, *A*_SiSi_ = *A*_OO_ = 1.5·10^−6^ kJ·(nm)^12^/mol, *B*_SiO_ = 4.2·10^−3^ kJ·(nm)^6^/mol, and *B*_SiSi_ = *B*_OO_ = 5·10^−5^ kJ·(nm)^6^/mol. The charge parameters of the oxygen and silicon atoms were initially chosen based on considerations arising from the Takada potential [19], and then the correctness of this choice was repeatedly confirmed by the accurate reproduction of the properties of silicon dioxide films deposited under various conditions [15,16]. Parameters of the Lennard-Jones (LJ) potential are the same for thermal and assisting oxygen atoms. The LJ-based potential used in the DESIL force field has high computational efficiency, which is important for the simulation of large clusters consisting of hundreds of thousands of atoms. The DESIL force field reliably reproduces the structural properties of fused silica and silicon dioxide films deposited under various conditions [15,16]. In addition, a deposited atom with a high initial kinetic energy of 10–100 eV can approach film atoms at a short distance of about 0.1 nm. At such distances, the Buckingham potential, which is used in the well-known BKS force field [20], may not work, since the repulsive exponential term is not compensated by the attractive term 1/r^6^. At the same time, the LJ potential works correctly at these interatomic distances. We assume that within the framework of the pairwise force fields with constant charges, similar to those used in the DESIL force field, we can consider the effect of the high kinetic energy of the assisting ions on the structure of the films. Note that film growth can also be simulated using force fields based on quantum chemical (QC) calculations, such as ReaxFF [21]. These force fields more accurately reproduce the formation of chemical bonds compared to empirical pairwise force fields of the DESIL type. However, the ability of QC-based force fields to work correctly with high-energy ions is not obvious, and the question of using such fields to simulate ion-assisted deposition requires a special separate study. In addition, the density functional modeling of SiO_2_ was performed in our recent works [22,23]. It was concluded that at the moment classical methods are more suitable for simulating film deposition, since when using quantum methods, the sizes of simulation clusters are still too small.

The GROMACS (Version 5.1.1) [24] program was used to perform the MD simulation. All calculations were carried out using the equipment of the shared research facilities of HPC computing resources at Lomonosov Moscow State University [25]. The 32 cores of Intel Haswell-EP E5-2697v3, 2.6 GHz processors are typically used in the parallel computations.

## 3. Results and Discussion

Let us start with an estimate of the ratio of the flux density of deposited atoms and assisting ions. The typical value of the deposition rate of SiO_2_ is about υ = 0.3–0.4 nm/s [4]. On the other hand, deposition rate can be expressed as follows:

υ = *dV*/(*Sdt*) = *dm*/(*ρSdt*) = *μdN*/(*N_A_ρSdt*) = *μn*/(*N_A_ρ*), (2)

where *μ =* 60 g/mol, *S,* *ρ* = 2∙10^3^ g/cm^3^*, N_A_* are the molar weight, surface area of the substrate, density of silicon dioxide film, and Avogadro number, respectively, *dN* is the number of deposited atoms per time interval *dt* to the substrate fragment with surface area *S*, *dV* is the deposited volume, and *n* is the flux density of deposited atoms:

*n* = *dN*/(*Sdt*), (3)

The value of *n* can be estimated using Equation (2):

*n =* υ*N_A_ρ*/*μ* ≈ 10^19^ 1/(s∙m^2^) (4)

Typical values of the current density *j* of assisting ions are several A/m^2^ [1], so the flux density of assisting ions is estimated as *n_i_* = *j*/*e* ≈ 10^19^ 1/(s∙m^2^). Thus, the flux densities of deposited atoms and assisting ions are close. In this work, the fraction of assisting ions is one of the simulation parameters and is specified as *f* = (*N*_O_/*N*_Si_)∙100%, where N_O_ is the number of assisting ions, and N_Si_ is the number of silicon atoms injected into the simulation box at step 2 (see Model Description section).

**Figure 2:**
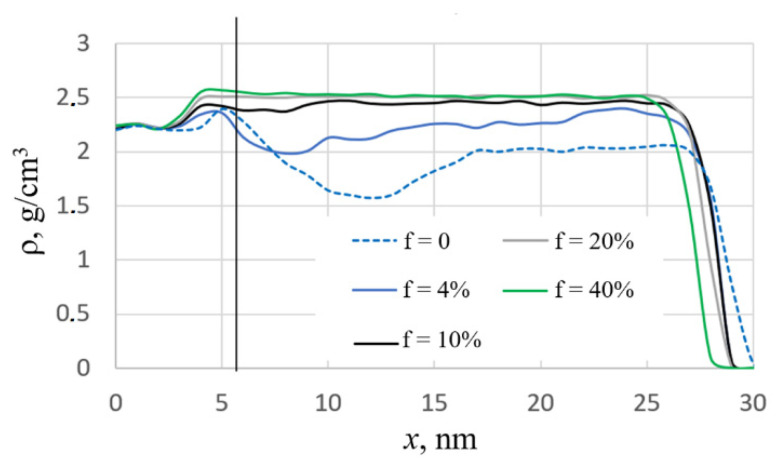
Density profiles of the deposited films: x is the coordinate of the film perpendicular to the substrate surface, x = 0 corresponds to the lower edge of the substrate, f is the fraction of assisting ions. The vertical line is the boundary between the substrate and the film, and energy of assisting ions E = 100 eV.

**Figure 3:**
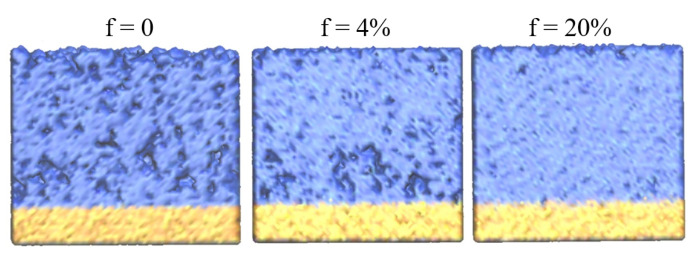
Atomistic structure of deposited films for various fractions of assisting oxygen atoms, f; energy of assisting ions E = 100 eV.

**Figure 4:**
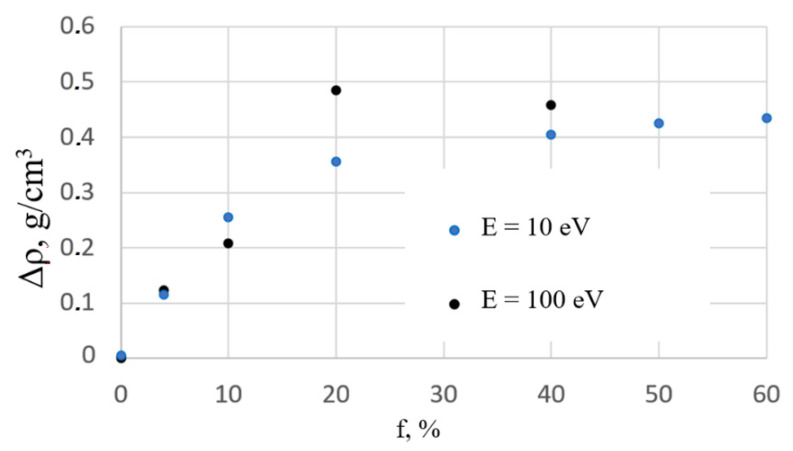
Dependencies of change in the film density Δρ on the fraction of assisting ions, f; E is the energy of assisting ions.

**Figure 5:**
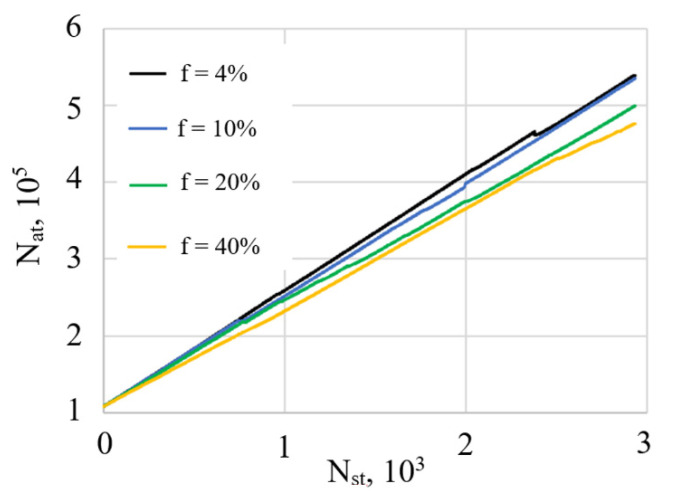
Dependence of the number of deposited atoms N_at_ on the number of deposition steps N_st_. E = 100 eV.

**Figure 6:**
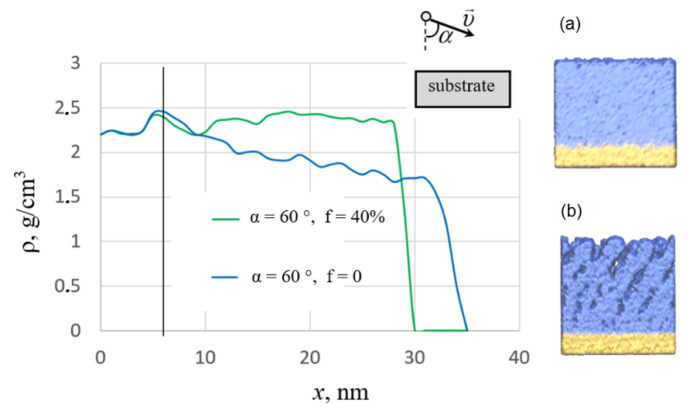
Density profiles of the IAD film deposited with ion assistance at α = 60°, f = 40%, and E = 100 eV (green line), and of the film obtained by the high-energy deposition at an angle α = 60° without ion assistance (blue line).

The results of MD simulation are presented in Figure 2, Figure 3, Figure 4, Figure 5 and Figure 6. The dependence of the density profiles on the fraction of assisting ions *f* is shown in Figure 2. As can be seen from the plots, deposition with assisting by high-energy ions leads to the film densification, which is consistent with experiments [4]. Low-energy deposition without ion assisting produces a porous film with a density of about 2.0 g/cm^3^ (blue dotted line in Figure 2). An increase in the fraction of assisting ions to about 10% is accompanied by an increase in the film density. A further increase in the *f* value does not have a noticeable effect on the density profile. The maximum value of film density ρ = 2.5 g/cm^3^ is close to that obtained in the previous simulations of the high-energy deposition of silicon dioxide films [14,15]. In addition, the ion-assisted deposition leads to the formation of films with smooth density profiles. To test the dependence of the results on the initial conditions, five runs were carried out with different sets of initial coordinates of the deposited atoms in the case of *f* = 10%. It was found that the differences in the density profiles were less than 0.05 g/cm^3^.

The concentration of the point defects is one of the key structural characteristics of optical films. It is found that the concentration *c* of non-bridging oxygen atoms O_1_ (low index is the coordination number) decreases monotonically with increasing *f* from *c*(O_1_) = 2.5% (*f* = 4%) to *c*(O_1_) = 1.7% (*f* = 40%). The concentration of three-coordinated silicon atoms shows a similar dependence: *c*(Si_3_) = 0.57% (*f* = 4%) and *c*(Si_3_) = 0.30% (*f* = 40%).

The statistic of n-membered rings in the films structure is calculated using the shortest-path analysis [26,27]. The main attention is paid to rings with a small number of atoms, n = 2 and 3, since the valence angles in these rings differ noticeably from the equilibrium values. This leads to the appearance of stresses in the structure. It is found that the fraction of the most stressed two-membered rings decreases from 1.2∙10^−3^ to 6∙10^−5^ with an increase in the fraction of the assisting ions from *f* = 4% to *f* = 40% at E = 100 eV. The fraction of three-membered rings decreases under these conditions from 5.0∙10^−2^ to 3∙10^−2^. Thus, an increase in *f* leads to a decrease in stresses in the film structure.

The surface roughness *R_h_* of the deposited films is calculated by the method described in [16]. As *f* increases from 4% to 10%, *R_h_* decreases from 0.57 nm to 0.34 nm. A further increase in *f* slightly affects the surface roughness. The value of *R_h_* = 0.34 nm is close to that obtained earlier in the simulation of high-energy deposition [16]. The decrease in the *R_h_* with an increase in the *f* value agrees with the previously obtained simulation results [10,12].

Visual analysis confirms the mentioned changes in the structure with an increase in the fraction of high-energy assisting oxygen ions (Figure 3). Micropores having different sizes and shapes are observed in the entire volume of the film deposited without ion assisting, *f* = 0. In the case of *f* = 4%, these micropores are observed mainly in the film layer near the substrate. The presence of micropores explains the local decrease in the density profile near the substrate (blue solid line in Figure 2). A further increase in *f* leads to the formation of a structure without these micropores.

The dependence of the change in film density on the fraction of assisted ions and their energy is shown in Figure 4. The density change is calculated as Δρ(*f*) = ρ(*f*) − ρ(*f* = 0), where ρ(*f* = 0) = 2.03 g/cm^3^ is the density of the film deposited without ion assisting. As can be seen from the plots in Figure 3, Δρ grows approximately linearly with *f* if *f* is less than 10%. Interestingly, in this interval of *f* values, Δρ(*f*) depends insignificantly on E. With a further increase in *f*, the growth of Δρ slows down and achieves the final value of 0.45 ÷ 0.5 g/cm^3^, which corresponds to a film density of 2.5 g/cm^3^. In the case of energy of the assisting ions E = 10 eV, the dependence of Δρ(*f*) is smoother than in the case of E = 100 eV. A similar dependence of density on the energy of assisting ions was found in the simulation of IAD of TiO_2_ films [12].

A change in the film density is accompanied by a corresponding change in the refractive index *n*, which for the silicon dioxide films can be estimated using the Gladstone–Dale equation [28] in the following form:

Δn = 0.21Δρ,(5)

Taking into account Equation (5) and the data shown in Figure 4, we find that Δn grows with the increase in *f* value and can reach 0.1. An increase in Δn by a few hundredths with an increase in the flow of the assisting ions was observed experimentally in [29].

The dependence of the film growth rate on the fraction of the assisting ions is shown in Figure 5. As can be seen from the plots in Figure 5, the number of the deposited atoms increases linearly with number of the injection steps. An increase in the fraction of assisting ions *f* leads to the decrease of the growth rate. This effect is due to an increase in the number of atoms reflected from the substrate and film with an increase in *f*. In addition, atoms of the film can be sputtered by high-energy assisting ions, which also reduces the growth rate. This trend was observed earlier in the atomistic simulations of IAD [9]. Thus, an increase in *f* leads to the films’ densification, but their growth rate somewhat decreases.

We also studied the dependence of the film structure on the angle between the direction of the assisting ions beam and the normal to the substrate (angle α in Figure 6). Under actual deposition conditions, the assisting ions flow covers a large area in the deposition chamber, and the directions of the ions flow in many areas differ significantly from the normal direction. It is known [30,31] that high-energy deposition at large angles α can form highly porous anisotropic films. Thus, it is important to check how ion assistance at large incidence angles affects the film structure.

In our studies, the direction of the assisting ions beam varies, while the deposited silicon atoms and thermal oxygen atoms move perpendicular to the substrate. Figure 6 shows results for the limiting case, when high-energy assisting ions move at an angle α = 60°. A dense film is formed without visible anisotropy of the structure (see green line and visual representation in box (a) in Figure 6). The density of this film is close to that obtained in high-energy normal deposition [14,15]. For comparison, Figure 6 also shows the results obtained in the case of high-energy deposition at an angle α = 60° (blue line and visual representation in box (b) without ion assisting. The formation of slanted columns and a significant decrease in the film density are clearly seen.

The optical anisotropy of the film can be quantitatively characterized by the difference Δ*n* of the main component of the refractive index tensor of the deposited films. In this work, the value of Δ*n* was calculated using the Bruggeman effective medium approach [32,33]. In this method, the anisotropic micropores are considered as identical ellipsoids with three different semi-axes, *a_x_*, *a_y_*, and *a_z_*. Values of *a_x_*, *a_y_*, and *a_z_* were calculated using the original approach [34], based on the Monte Carlo method. It was found that even in the limiting case of α = 60°, f = 40%, and E = 100 eV, the value of Δ*n* was less than 0.007, which means that the film can be considered isotropic.

## 4. Conclusions

In this work, the classical molecular dynamics simulation of ion-assisted deposition of silicon dioxide films was used to systematically study the main structural parameters of the deposited films. The energy of the deposited silicon and oxygen atoms was 0.1 eV, which corresponds to the thermal evaporation deposition process. The energy of the assisting oxygen ions was taken to be 100 eV.

It was found that an increase in the flow of assisting ions to approximately 10% of the flow of deposited silicon atoms leads to an increase in film density up to 0.5 g/cm^3^. A further increase in the flux density of the assisted atoms insignificantly affects the film density. The increase in film density is associated with the disappearance of micropores that exist in the films deposited without ion assistance. The increase in the refractive index associated with the increase in density reached 0.1. The concentration of the point defects, which affect the optical properties, and stressed structural rings with two and three silicon atoms decreased with an increase in the flux of assisting ions. In addition, the ion assistance noticeably reduced surface roughness.

The film growth rate somewhat decreased with an increase in the assisting ions flux. The anisotropy of films deposited under assisting ions beam directed at an angle of 60° to the normal to the substrate was estimated using the Bruggeman effective medium approach. It was found that the differences between the main components of the refractive index tensor are insignificant.

## Data Availability

Not applicable.
